# Comparing three cardiothoracic ratio measurement techniques and creating multivariable scoring system to predict Bart’s hydrops fetalis at 17–22 weeks’ gestation

**DOI:** 10.1038/s41598-024-59719-8

**Published:** 2024-04-17

**Authors:** Sanitra Anuwutnavin, Patsawee Rangseechamrat, Nalat Sompagdee, Pornpimol Ruangvutilert, Sommai Viboonchard

**Affiliations:** Department of Obstetrics and Gynaecology, Faculty of Medicine Siriraj Hospital, Mahidol University, 2 Prannok Road, Bangkoknoi, Bangkok, 10700 Thailand

**Keywords:** Ultrasonography, Cardiothoracic ratio, Middle cerebral artery-peak systolic velocity, Placental thickness, Hemoglobin Bart’s disease, Prediction, Multivariable scoring system, Haematological diseases, Ultrasonography

## Abstract

To assess the diagnostic performance of three cardiothoracic (CT) ratio techniques, including diameter, circumference, and area, for predicting hemoglobin (Hb) Bart’s disease between 17 and 22 weeks’ gestation, and to create a multivariable scoring system using multiple ultrasound markers. Before invasive testing, three CT ratio techniques and other ultrasound markers were obtained in 151 singleton pregnancies at risk of Hb Bart’s disease. CT diameter ratio demonstrated the highest sensitivity among the other techniques. Significant predictors included CT diameter ratio > 0.5, middle cerebral artery-peak systolic velocity (MCA-PSV) > 1.5 multiples of the median, and placental thickness > 3 cm. MCA-PSV exhibited the highest sensitivity (97.8%) in predicting affected fetuses. A multivariable scoring achieved excellent sensitivity (100%) and specificity (84.9%) for disease prediction. CT diameter ratio exhibited slightly outperforming the other techniques. Increased MCA-PSV was the most valuable ultrasound marker. Multivariable scoring surpassed single-parameter analysis in predictive capabilities.

## Introduction

Alpha thalassemia is a red blood cell disorder common in Southeast Asia, the Middle East, China, and Africa. Hemoglobin (Hb) Bart’s disease is a severe form that causes fetal tissue hypoxia and severe anemia. Hydrops fetalis usually occurs in the second half of pregnancy and can lead to stillbirth and maternal complications^1–5^. Nonetheless, Hb Bart's disease is no longer universally regarded as a fatal disorder. The increasing number of survivors can be attributed to intrauterine transfusions and neonatal intensive care^2,6,7^. Despite these improvements, there remains a high incidence of associated congenital abnormalities and growth impairment among survivors^2,6^. Some individuals may experience neurodevelopmental delays^2,6^. Long-term transfusions and iron chelation are necessary for most patients following hematopoietic stem cell transplantation, which is currently the only definitive cure^2,7,8^. In utero stem cell transplantation or gene therapy/editing shows promise as another approach, but it is still in the clinical trial stage^7^.

Prenatal diagnosis of Hb Bart’s disease involves invasive procedures such as chorionic villus sampling, amniocentesis, and cordocentesis. However, these procedures carry risks of fetal loss^9,10^. To minimize these risks, serial ultrasonography is being explored as a noninvasive alternative approach^11^. Previous studies have identified ultrasound markers, including enlarged fetal heart or increased cardiothoracic (CT) ratio, elevated middle cerebral artery-peak systolic velocity (MCA-PSV), placentomegaly, and hepatosplenomegaly, that hold potential for predicting fetal Hb Bart’s disease^3,12–15^.

Fetal CT ratios can be computed using three methods: CT diameter (CTD), CT circumference (CTC), or CT area (CTA)^16^. Prior studies indicate a slight increase in fetal CT ratios with advancing gestational age (GA)^16–18^. However, only the CTD ratio is commonly employed to assess fetal cardiomegaly for detecting Hb Bart’s disease^11–14,19,20^. Limited research exists on evaluating fetal CTC and CTA ratios as sonographic predictors for Hb Bart’s disease. Therefore, this study aimed to determine optimal cutoff values and assess the diagnostic performance of CTD, CTC, and CTA ratios for identifying Hb Bart’s disease at 17–22 gestational weeks. Additionally, we evaluated midpregnancy ultrasonographic features such as MCA-PSV and placental thickness (PT). Furthermore, our secondary objective was to develop a multivariable scoring system incorporating multiple ultrasound markers to enhance disease prediction accuracy.

## Materials and methods

This retrospective and prospective cohort study was conducted on pregnant women at risk of fetal Hb Bart’s disease who underwent second-trimester ultrasound examination and invasive prenatal testing at the Maternal-Fetal Medicine (MFM) Unit of Siriraj Hospital, Bangkok, Thailand, from June 2018 to November 2022. Retrospective data from 129 cases (June 2018–November 2021) and prospective data from 22 cases (December 2021–November 2022) were collected to achieve an adequate sample size. The institutional review board of Siriraj Ethics Committee authorized the research protocol (approval no. Si-897/2021), and written informed consent was obtained from all prospective study participants. The study was performed in accordance with the Declaration of Helsinki.

The study included data from singleton pregnant women who were at risk of carrying a fetus with Hb Bart’s disease based on a maternal and paternal thalassemia blood test screening (alpha thal-1 trait, Hb H, and Hb H Constant Spring/Pakse disease). These women underwent invasive diagnostic procedures (amniocentesis or cordocentesis) for fetal alpha DNA testing or hemoglobin typing between 17 and 22 weeks of gestation. Exclusion criteria were fetal structural and/or functional cardiac anomalies, beta thalassemia major, fetal infection, other causes of fetal anemia, major extracardiac defects, chromosomal abnormalities, and abnormal fetal growth. Women with unknown fetal alpha globin gene analysis results and/or Hb typing, or those lost to follow-up for postprocedural outcomes, were excluded.

**Figure 1:**
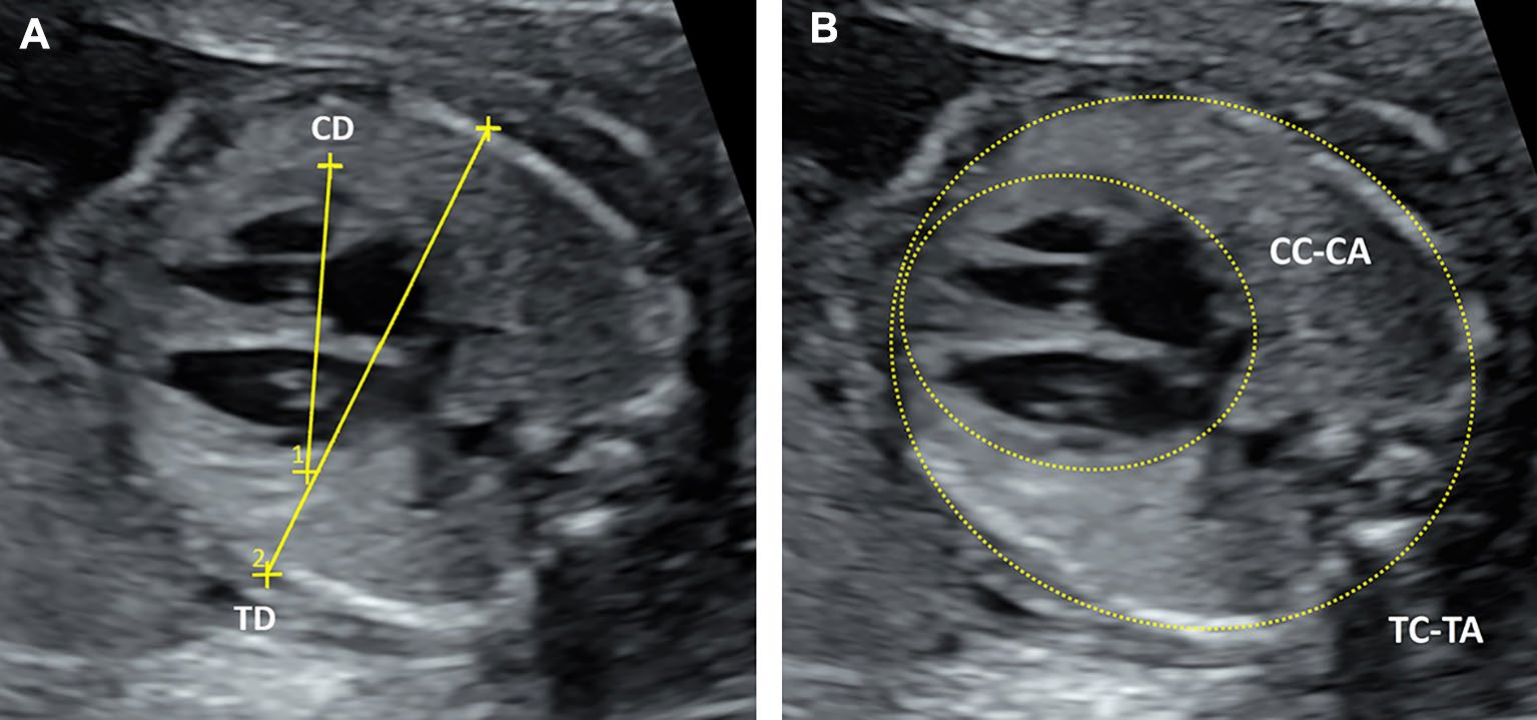
Fetal thorax at the level of four‐chamber view during end‐diastole showing (**A**) measurement of fetal cardiothoracic diameter ratio and (**B**) cardiothoracic circumference and area ratios by automated ellipse method. *CC‐CA* cardiac circumference and cardiac area, *CD* cardiac diameter, *TC‐TA* thoracic circumference and thoracic area, *TD* thoracic diameter.

Before prenatal genetic testing, ultrasound examinations were performed by an MFM staff member or an MFM fellow using an abdominal 1–5-MHz curvilinear transducer (Voluson E10, E8, or E6; GE Medical Systems, Chicago, IL, USA). The CTD, CTC, and CTA ratios were measured on cross-sectional images of the fetal chest at the level of the 4-chamber view during end-diastole (Fig. 1A,B). The measurement techniques were described in our previous study^16^. For each ratio, measurements were repeated three times, with the average calculated from recorded video clips or still images. Intraobserver and interobserver variabilities were not assessed, as our previous study reported good to excellent agreement for the measurements^16^.

Additional evaluated ultrasonographic features were MCA-PSV, PT, soft markers of aneuploidy, and signs of hydropic changes. MCA-PSV measurements followed the technique proposed by the Society for Maternal–Fetal Medicine in 2015^21^, with the maximum point of the MCA Doppler waveform selected from still images. MCA-PSV values were expressed as multiples of the median (MoM) by dividing them by the Thai nomogram medians for specific gestational ages reported by Tongsong et al.^22^ PT was measured by placing the transducer perpendicularly to the placenta and averaging the longitudinal and transverse plane measurements of its center^23,24^. “Hydrops fetalis” was defined as the presence of at least two abnormal fluid collections (pleural and pericardial effusions, ascites, or fluid accumulation with anasarca)^25^. All ultrasound findings were obtained before receiving the invasive testing results. Hb Bart’s disease confirmation relied on fetal polymerase chain reaction for alpha globin gene analysis from amniocentesis or Hb typing using high-performance liquid chromatography and alpha-globin gene analysis from cordocentesis.

### Statistical analysis

Statistical analyses were conducted using IBM SPSS Statistics, version 29 (IBM Corp, Armonk, NY, USA), and STATA 17.0 software (StataCorp LLC, College Station, TX, USA). Demographic data are presented as numbers (percentages) and means ± standard deviations. The normal distribution was assessed using the Shapiro–Wilk test. Unpaired *t*-tests compared continuous variables between fetuses affected and unaffected by Hb Bart’s disease. Categorical variables were analyzed using Pearson’s chi-square or Fisher’s exact test. Receiver operating characteristic (ROC) curves determined optimal cutoff values for ultrasonographic markers. Differences in areas under the curve (AUCs) were assessed using the Z-test. Diagnostic parameters (sensitivity, specificity, positive predictive value, negative predictive value, accuracy, false positives [FPs], and false negatives [FNs]) were reported. Univariate analysis identified prenatal predictors associated with Hb Bart’s disease, and multiple logistic regression analysis evaluated the interdependence of significant variables. The most discriminative independent variables were selected. Statistical significance was set at *P* < 0.05. A multivariable sonographic scoring system was developed, and various cutoffs were evaluated for diagnostic performance. Discriminatory power was assessed using AUC analysis, and calibration plots compared the observed and expected outcome frequencies. The Brier score verified probability forecast accuracy, with a score close to zero indicating optimal model performance. The Hosmer–Lemeshow test assessed calibration, with *P* > 0.05 indicating good calibration.

## Results

During the study period, 151 pregnancies at risk were eligible. Among them, 45 fetuses (29.8%) were diagnosed with Hb Bart’s disease. Frank Hb Bart’s hydrops fetalis and Hb H or Hb H Constant Spring/Pakse disease were detected in 9 cases (6.0%) and 10 cases (6.6%), respectively, the latter being included in the unaffected group. Table 1 shows the baseline characteristics of affected and unaffected pregnancies, including maternal age, parity, GA at ultrasound examination, body mass index, underlying maternal disease, types of parental alpha and beta globin gene analysis, and invasive prenatal diagnostic techniques. No significant differences were found between the groups, except for a significantly higher cordocentesis rate in the affected group (64.4%) than in the unaffected group (0%; *P* < 0.001). Regarding postprocedural outcomes, all affected pregnancies were safely terminated vaginally, and no procedure-related pregnancy losses occurred.

**Table 1:** The demographic characteristics of fetuses with and without hemoglobin Bart’s disease.

Demographic data	Fetal Hb Bart's disease		*P*-values
	Affected (n = 45)	Unaffected (n = 106)	
Maternal age (years)	30.00 ± 6.01	29.58 ± 4.87	0.650^a^
Parity			0.871^b^
Nulliparous	24 (53.3)	55 (51.9)	
Multiparous	21 (46.7)	51 (48.1)	
Gestational age at ultrasound (weeks)	19.13 ± 1.51	18.70 ± 0.90	0.080^a^
Body mass index	23.46 ± 4.00	23.37 ± 4.08	0.908^a^
Maternal underlying diseases			0.083^b^
No	43 (95.6)	102 (96.2)	
Chronic hypertension	2 (4.4)	0 (0)	
Gestational diabetes mellitus	0 (0)	1 (0.9)	
Others	0 (0)	3 (2.8)	
Maternal alpha globin gene analysis result			0.672^b^
Alpha thal-1 trait	39 (86.7)	89 (84.0)	
Hb H or Hb H CS/PS disease	6 (13.3)	17 (16.0)	
Paternal alpha globin gene analysis result			0.194^b^
Alpha thal-1 trait	42 (93.3)	91 (85.8)	
Hb H or Hb H CS/PS disease	3 (6.7)	15 (14.2)	
Maternal beta globin gene analysis result			0.910^b^
Normal	31 (68.9)	74 (69.8)	
Abnormal	14 (31.1)	32 (30.2)	
Paternal beta globin gene analysis result			0.120^b^
Normal	38 (84.4)	77 (72.6)	
Abnormal	7 (15.6)	29 (27.4)	
Types of invasive procedure			< 0.001^b^
Amniocentesis	16 (35.6)	106 (100.0)	
Cordocentesis	29 (64.4)	0 (0)	

Data are presented as number (percent), number, and mean ± SD where applicable.

*CS* Constant Spring, *Hb* hemoglobin, *PS* Pakse, *thal* thalassemia.

^a^Unpaired t-test.

^b^Pearson χ^2^ or Fisher’s exact test.

Measuring fetal CT ratios using all three methods was technically feasible. Fetuses with Hb Bart’s disease exhibited significantly higher mean CTD, CTC, and CTA ratios than unaffected fetuses (0.58 ± 0.06 vs 0.45 ± 0.04, 0.60 ± 0.05 vs 0.50 ± 0.03, and 0.36 ± 0.06 vs 0.24 ± 0.03, respectively; all *P* < 0.001). The affected group also had significantly higher mean MCA-PSV and PT measurements than the unaffected group (2.01 ± 0.27 vs 1.26 ± 0.24 MoM and 3.60 ± 1.01 vs 2.56 ± 0.54 cm, respectively; all *P* < 0.001). Among the second-trimester soft markers, fetuses with Hb Bart’s disease demonstrated a significantly higher rate of hyperechoic bowel (*P* < 0.001), while no significant differences were observed in the remaining markers (*P* = 0.322–1.000).

**Figure 2:**
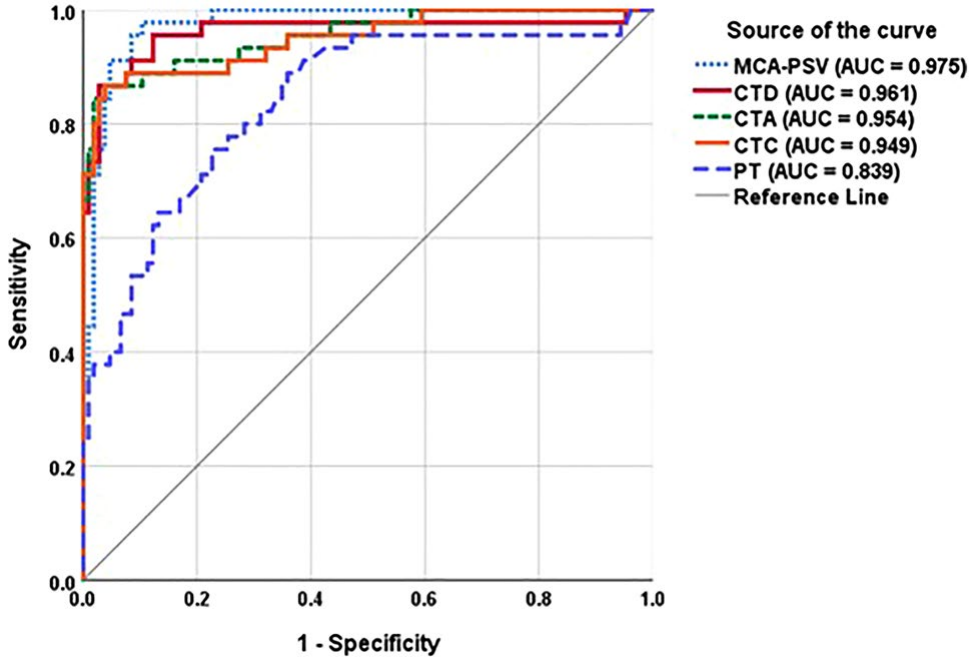
Receiver-operating-characteristics curves of three fetal cardiothoracic ratio techniques, middle cerebral artery-peak systolic velocity, and placental thickness for prediction of fetal hemoglobin Bart’s disease. *AUC* area under the curve, *CTA* cardiothoracic area ratio, *CTC* cardiothoracic circumference ratio, *CTD* cardiothoracic diameter ratio, *MCA-PSV* middle cerebral artery-peak systolic velocity, *PT* placental thickness.

Based on ROC curve analysis (Table 2 and Fig. 2), the optimal cutoff values for differentiating affected and unaffected fetuses were as follows: CTD ratio 0.5 (AUC 0.961; 95% CI 0.917–1.000); CTC ratio 0.54 (AUC 0.949; 95% CI 0.908–0.990); and CTA ratio 0.29 (AUC 0.954; 95% CI 0.917–0.992). While the CTD ratio exhibited slightly better AUC and sensitivity (91.1%), there were no significant differences between the AUCs of the three methods. Using a cutoff of 1.5 MoM for MCA-PSV provided the best discrimination (AUC 0.975; 95% CI 0.954–0.996) with the highest AUC and sensitivity (97.8%). Conversely, a PT cutoff of 3 cm had the lowest AUC and sensitivity (AUC 0.839; 95% CI 0.766–0.911; sensitivity 73.3%). All three CT ratio measurement techniques and MCA-PSV had significantly higher AUCs than PT (*P* < 0.001–0.009). However, there were no significant differences in the AUCs of the three fetal CT ratios and MCA-PSV (*P* = 0.280–0.558).

**Table 2:** Diagnostic performance of three cardiothoracic ratio techniques, middle cerebral artery-peak systolic velocity, placental thickness, and echogenic bowel in predicting fetal hemoglobin Bart’s disease.

Sonographic markers	Cut-off	Affected (n = 45)	Unaffected (n = 106)	Sensitivity	Specificity	PPV	NPV	Accuracy	FP	FN
CT ratios										
CTD	> 0.50	41 (78.8)	11 (21.2)	91.1	89.6	78.8	96	90.1	10.4	8.9
CTC	> 0.54	40 (74.1)	14 (25.9)	88.9	86.8	74.1	94.8	87.4	13.2	11.1
CTA	> 0.29	39 (83)	8 (17)	86.7	92.5	83	94.2	90.7	7.5	13.3
MCA-PSV	> 1.5 MoM	44 (77.2)	13 (22.8)	97.8	87.7	77.2	98.9	90.7	12.3	2.2
PT	> 3 cm	33 (57.9)	24 (42.1)	73.3	77.4	57.9	87.2	76.2	22.6	26.7
Echogenic bowel	Present	8 (17.8)	1 (0.9)	17.8	99.1	88.9	73.9	74.8	0.9	82.2

Data are presented as number (percent), and percent where applicable.

*cm* centimeter, *CT* cardiothoracic, *CTA* cardiothoracic area ratio, *CTC* cardiothoracic circumference ratio, *CTD* cardiothoracic diameter ratio, *FN* false negative, *FP* false positive, *MCA-PSV* middle cerebral artery-peak systolic velocity, *MoM* multiple of the median, *NPV* negative predictive value, *PPV* positive predictive value, *PT* placental thickness.

We excluded 9 cases of frank hydrops fetalis from the cohort and repeated the statistical analysis. These fetuses exhibited distinct signs of Hb Bart’s disease and may not benefit from predictive sonographic parameters. However, the optimal cutoffs for each ultrasound marker remained unchanged. MCA-PSV maintained the highest sensitivity (97.2%), followed by the CTD ratio (94.4%), CTC ratio (94.4%), CTA ratio (91.7%), and PT (69.4%) (data not shown).

By using multiple logistic regression analysis, significant ultrasonographic parameters and their cutoff values were identified: CTD ratio > 0.5 (*P* < 0.001), MCA-PSV level < 1.5 MoM (*P* < 0.001), and PT measurement > 3 cm (*P* = 0.033). A multivariable scoring system was then developed to predict fetal Hb Bart’s disease. Variables included in the system were selected based on statistical significance and assigned values corresponding to their coefficients. An increased PT > 3 cm received a value of 1, a CTD ratio > 0.5 received a value of 2, and an elevated MCA-PSV MoM > 1.5 received a maximum value of 2.5. Total scores ranged from 0 to 5.5 (Table 3).

**Table 3:** Development of sonographic scoring system for prediction of fetal hemoglobin Bart’s disease derived from multivariate logistic regression analysis (total scores = 5.5).

Sonographic variables	Coefficient (95% CI)	*P*-values	Score points
CTD > 0.50	4.65 (2.33–6.97)	< 0.001	2
MCA-PSV > 1.5 MoM	5.76 (2.91–8.62)	< 0.001	2.5
PT > 3 cm	2.43 (0.20–4.67)	0.033	1

*CI* confidence interval, *cm* centimeter, *CTD* cardiothoracic diameter ratio, *MCA-PSV* middle cerebral artery-peak systolic velocity, *MoM* multiple of the median, *PT* placental thickness.

Table 4 presents the diagnostic abilities of different cutoffs of the sonographic scoring system for identifying fetuses with Hb Bart’s disease. Two cutoffs were assessed. A threshold score of ≥ 2.5 yielded 100% sensitivity, 84.9% specificity, 89.4% accuracy, 15.1% FP, and 0% FN for disease detection. Alternatively, a threshold score of ≥ 3 achieved higher specificity (90.6%), accuracy (92.7%), and FP (9.4%) but slightly lower sensitivity (97.8%) and FN (2.2%).

**Table 4:** Predictive properties of differing cutoff values of the sonographic scoring system in predicting fetal hemoglobin Bart’s disease.

Cutoffs	Sensitivity (%)	Specificity (%)	PPV (%)	NPV (%)	Accuracy (%)	FP (%)	FN (%)
≥ 2.5	100	84.9	73.8	100	89.4	15.1	0
≥ 3	97.8	90.6	81.5	99	92.7	9.4	2.2

*FN* false negative, *FP* false positive, *NPV* negative predictive value, *PPV* positive predictive value.

**Figure 3:**
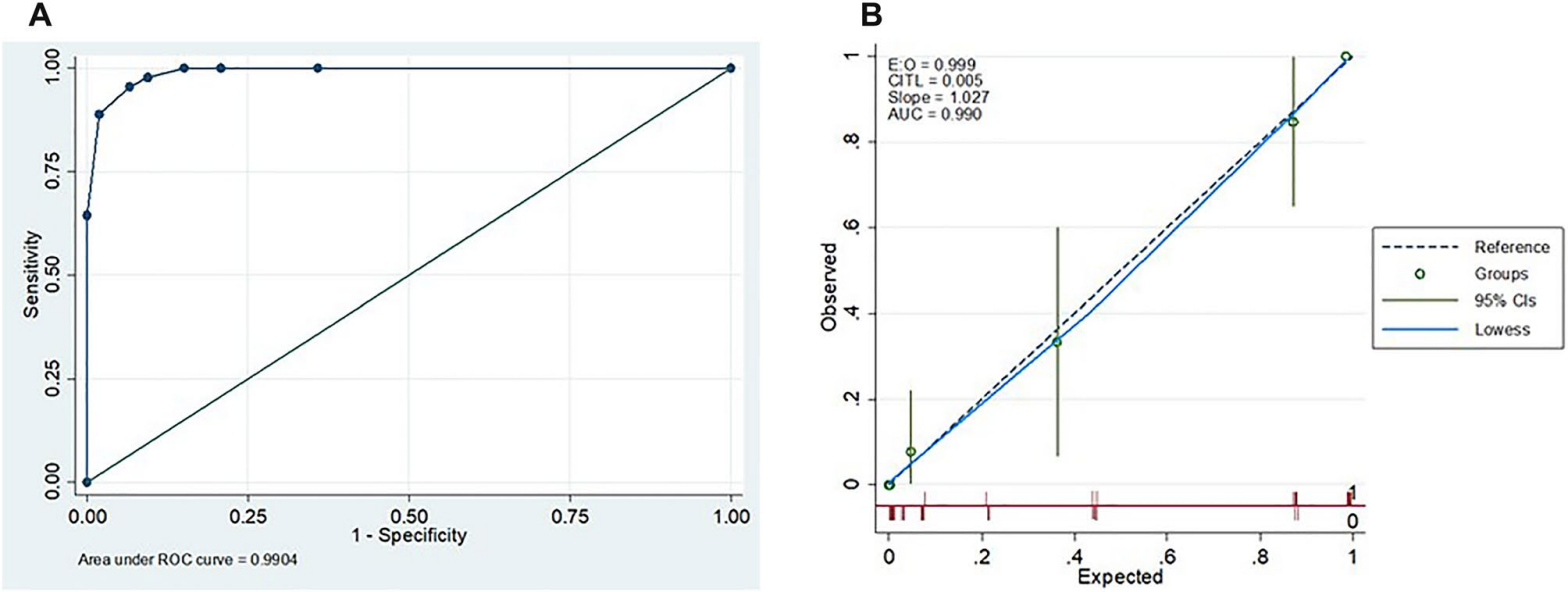
(**A**) Receiver-operating-characteristics curves of the sonographic scoring system for discriminating between fetuses with and without hemoglobin Bart’s disease (area under the curve = 0.99). (**B**) The calibration plot of the scoring system demonstrated great consistency between the predicted and observed outcome probabilities.

The multivariable scoring system was internally validated using the AUC and calibration plot (Fig. 3). The system demonstrated excellent discriminatory ability, with an AUC of 0.990 (95% CI 0.980–1.000; *P* < 0.001). The calibration plot showed excellent fit (slope 1.027; intercept 0.005). The Hosmer–Lemeshow test yielded a *P* value of 0.829, indicating good calibration, and the Brier score was 0.035, signifying optimal calibration.

## Discussion

Fetuses with Hb Bart’s disease exhibit cardiovascular adaptations to compensate for anemia and prevent tissue hypoxia. The initial structural change is cardiac chamber enlargement, increasing blood volume and cardiac output for optimal tissue oxygenation^3,5,26^. The CTD ratio, representing fetal cardiomegaly, has shown excellent predictive ability for Hb Bart’s disease^8,11–14,19,26,27^. Our study confirmed the effectiveness of all three fetal CT ratio measurement techniques in distinguishing affected and unaffected pregnancies. While the CTD ratio demonstrated slightly higher sensitivity, there were no significant differences in overall diagnostic accuracy (Fig. 2).

Comparisons with previous publications are summarized in Table 5. Our study’s optimal cutoff for the CTD ratio aligned with previous reports^8,11–14,19,26^. The best cutoff for each fetal CT ratio method surpassed or equaled the 95th centile of GA-specific references for normal Thai fetuses, indicating existing fetal cardiomegaly^16^. Regarding sensitivity, our CTD ratio was slightly lower than that in other studies. This discrepancy may be attributed to the inclusion of 9 hydropic fetuses, some of whom presented with marked pericardial or pleural effusion. Consequently, fetal thoracic diameter appeared longer, resulting in decreased CTD values and potentially reduced sensitivity^8,19^. Upon excluding all cases of hydrops fetalis, the new sensitivity for the fetal CTD ratio reached 94.4%, similar to earlier studies.

MCA-PSV Doppler assessment is recommended as the primary method for detecting fetal anemia, regardless of etiology^21^. Our study found that MCA-PSV had the highest sensitivity (97.8%) for identifying Hb Bart’s disease, which was moderately higher than previous reports^13,28–30^. This difference may be attributed to using advanced ultrasound technology with high-resolution probes for 2-dimensional and Doppler ultrasonography and improved MCA-PSV assessment by examiners. We utilized Thai nomograms for median MCA-PSV values^22^, which appeared lower than those used in a study on the Caucasian population by Mari et al.^31^ This could result in an increased detection rate of affected fetuses compared to studies using normative data from different populations^13^. Furthermore, it has been observed that most affected fetuses (92.3%) develop some degree of anemia in midgestation, often presenting with elevated MCA-PSV before 23 weeks of gestation^20,27,32^. Therefore, elevated MCA-PSV serves as a direct indicator of fetal anemia, the disease’s pathophysiology, during the mid-second trimester.

**Table 5:** Comparison of the results of the cutoff and predictive values of sonographic markers of previously published studies and the present study.

Publications	Sample size	GA (weeks)	Cut-offs		Sensitivity	Specificity	PPV	NPV
CTD								
Lam et al. (1997)^11^	75	17–18	≥ 0.50		100	93	80	100
Tongsong et al. (1999)^12^	345	18–21	≥ 0.50		98.6	98.9	95.8	99.6
Tongsong et al. (2004)^14^	488	18–21	> 0.50		95	96.1	86.4	98.7
Leung et al. (2006)^19^	832	16–17	≥ 0.50		96.7	94.1	–	–
		18–20	≥ 0.52					
Leung et al. (2010)^13^	410	16–17	≥ 0.50		100.0	94.8	61.5	100
		18–20	≥ 0.52					
Wanapirak et al. (2017)^20^	275	15–23	≥ 0.50		100	91.9	79	100
Chankhunaphas et al. (2021)^26^	1,717	18–20	> 0.50		93.1	94	–	–
		21–23	> 0.51		98	91.1	–	–
Hui et al. (2022)^8^	304	16–17	≥ 0.50		93.8	94.1	65.2	99.2
		18–20	≥ 0.52					
Present study	151	17–22	> 0.50		91.1	89.6	78.8	96
MCA-PSV								
Srisupundit et al. (2009)^28^	91	18–22	> 1.5 MoM		85	100	100	95.9
Leung et al. (2010)^13^	79	16–20	> 1.5 MoM		64.3	98.6	90	93.3
Luewan et al. (2011)^30^	298	18–22	> 1.5 MoM		91	98.2	94.7	96.9
Tongprasert et al. (2019)^29^	142	18–22	> 1.5 MoM		84.8	89	79.6	92
Present study	151	17–22	> 1.5 MoM		97.8	87.7	77.2	98.9
PT								
Ghosh et al. (1994)^24^	75	18–21	> + 2 SD		100	96	–	–
Tongsong et al. (1999)^23^	345	18–21	> 3 cm		88.6	90.2	78.5	96.9
Tongsong et al. (2004)^14^	488	18–21	> 3 cm		74	96.1	83.1	93.5
Present study	151	17–22	> 3 cm		73.3	77.4	57.9	87.2
Echogenic bowel								
Lam et al. (1999)^33^	126	12–24	Present		30.56	100	100	88.64
Hui et al. (2022)^8^	304	12–20	Present		12.5	98.9	57.1	90.6
Present study	151	17–22	Present		17.8	99.1	88.9	73.9
Combined ultrasonographic markers								
Leung et al. (2010)^13^	79	16–20	CTD or MCA-PSV		100	69	54.2	100
			CTD and MCA-PSV		61.5	100	100	98.7
Harn-a-morn et al. (2022)^27^	237	12–24	CTD or MCA-PSV		100	89.1	72.6	100
Present study	151	17–22	CTD and MCA-PSV and PT	≥ 2.5	100	84.9	73.8	100
				≥ 3	97.8	90.6	81.5	99

*cm* centimeter, *CTD* cardiothoracic diameter ratio, *GA* gestational age, MCA-PSV middle cerebral artery-peak systolic velocity, *MoM* multiple of the median, *NPV* negative predictive value, *PPV* positive predictive value, *PT* placental thickness, *SD* standard deviation.

Comparing optimal cutoffs for MCA-PSV, most previous studies, including ours, used values above 1.5 MoM^13,28–30^. Tongprasert et al.^29^ proposed a cutoff of 1.3 MoM to detect even mildly anemic-affected fetuses during midpregnancy, achieving exceptional sensitivity (100%) but moderate specificity (69.2%). If we adopted the 1.3 MoM cutoff, our results aligned with the other, demonstrating a high sensitivity of 100% but a lower specificity of 64.2% (data not shown).

Among the midtrimester ultrasound parameters, MCA-PSV exhibited the highest sensitivity, followed by the CTD ratio and PT (Table 2). However, there were no significant differences in the AUCs of the CTD ratio and MCA-PSV, indicating similar diagnostic accuracy for detecting fetal Hb Bart’s disease. PT showed lower sensitivity and specificity than the CTD ratio and MCA-PSV, consistent with previous studies^11,14,19,23^. This can be attributed to the fact that placental thickening becomes more apparent later in gestation than an increase in the CTD ratio or MCA-PSV^3,5,14,27^. While some studies have suggested that the CTD ratio is superior to MCA-PSV as a single screening marker throughout gestation^8,13,26,27^, our findings demonstrate that the CTD ratio and MCA-PSV are equally effective predictors, particularly in the mid-second trimester.

Regarding the association between soft markers and fetal Hb Bart’s disease, we observed a significantly higher prevalence of echogenic bowel among affected pregnancies, consistent with other studies^8,33^. This could be attributed to bowel hypoperistalsis or bowel wall edema resulting from severe fetal anemia and hypoxia^33^. However, due to this parameter’s subjective evaluation and limited sensitivity, we did not include it in the multivariable scoring system.

Our study developed a multivariable scoring system using sonographic evaluation to noninvasively predict fetal Hb Bart’s disease. The system included several ultrasound parameters with high diagnostic accuracy and assigned scores based on their significant association with the disease. Unlike previous articles that relied on a single marker or a combination of CTD and MCA-PSV (Table 5), our approach utilized a broader set of markers. Our and other data also revealed that the combination of the CTD ratio and/or MCA-PSV achieved the highest detection rate (100%), outperforming the use of any individual marker^13,27,28^.

The increase in MCA-PSV above 1.5 MoM was the most significant marker for distinguishing affected and unaffected fetuses, with the highest sensitivity (97.8%) and accuracy (90.7%). An MCA-PSV level exceeding 1.5 MoM, assigned a score of 2.5, was the best single parameter for predicting Hb Bart’s disease (Table 4). In cases where this finding was absent, detecting Hb Bart’s disease required positive results for the remaining two variables in the scoring system. Including multiple markers in the system improved its sensitivity and reduced FNs compared to relying solely on MCA-PSV levels. This was evident in one case where Hb Bart’s disease was genetically confirmed despite an MCA-PSV level of only 1.41 MoM.

The sonographic scoring system serves as a helpful screening tool for differentiating high-risk fetuses requiring invasive procedures from low-risk fetuses suitable for serial ultrasound surveillance. A threshold score of ≥ 2.5 achieved a 100% detection rate for fetal Hb Bart’s disease. However, there were drawbacks, with 15.1% of unaffected fetuses identified as positive, necessitating invasive evaluation (Table 4). Notably, 3 cases (30%) of fetal Hb H disease had only an MCA-PSV MoM value above the cutoff of 1.5, signifying FP results. Alternatively, defining a rating score of ≥ 3 reduced the FP rate to 9.4% but increased the FN rate to 2.2%.

In the clinical implementation of the multivariable scoring system, a score ≥ 2.5 would warrant invasive diagnostic procedures. Conversely, cases with a score < 2.5 can be managed with regular ultrasound monitoring every 2–4 weeks until 24 weeks' gestation to assess sonographic markers and hydropic signs. Studies suggest that additional ultrasound examinations beyond 23–24 weeks gestation may be unnecessary if no positive ultrasound features or hydropic changes are identified^3,20,27^. If a rating score of ≥ 2.5 and/or hydropic signs emerged during follow-up ultrasounds, confirmatory prenatal diagnosis through invasive genetic testing was pursued. If the scoring system indicates abnormalities that cannot be explained by Hb Bart’s disease or other causes of cardiomegaly, fetal echocardiography is recommended for a more precise and detailed examination to evaluate the cardiac structures.

The study’s strengths lie in measuring all ultrasound parameters during invasive prenatal diagnosis, ensuring accurate fetal diagnosis confirmed by molecular genetic testing. This allowed for precise classification of fetuses with Hb Bart’s disease versus Hb H or Hb H Constant Spring/Pakse disease and the perception of differences in the sonographic markers of these conditions, such as increased MCA-PSV without cardiomegaly in 30% of fetuses with Hb H or Hb H Constant Spring/Pakse disease. Additionally, we determined optimal cutoff values, compared the diagnostic accuracy of different CT ratio techniques and other ultrasound findings in predicting Hb Bart’s disease, and developed a multivariable scoring system with high sensitivity and specificity for clinical decision-making in invasive prenatal diagnosis.

However, our study has several limitations. First, the findings are specific to the mid-second trimester and cannot be generalized to other gestational periods, particularly the first trimester. Second, some retrospective study patients had limitations in image quality. Third, the sensitivity and specificity of the markers for Hb Bart’s disease may vary when assessed by general practitioners, as MFM trainees and specialists performed our assessments. Fourth, the automated ellipse method might exhibit double errors in both CTC and CTA ratios compared to the CTD ratio. Results may differ when using manual tracing. Finally, given the limited sample size in our study, further research is necessary to evaluate the diagnostic accuracy of all three fetal CT ratio methods. Additionally, assessing the effectiveness, obstacles, and cost-effectiveness of the multivariable scoring approach in routine general practice is warranted.

## Conclusions

Our study found that all three fetal CT ratio measurement techniques effectively identified Hb Bart’s disease, with the CTD ratio showing slightly better sensitivity. Additionally, elevated MCA-PSV above 1.5 MoM demonstrated the highest sensitivity and accuracy for predicting affected fetuses. The multivariable scoring system enhanced sensitivity and specificity compared to individual ultrasound parameters alone, making it suitable for clinical practice. This noninvasive approach is a valuable tool that improves prenatal detection of Hb Bart’s disease, reduces medical costs, and avoids risks associated with invasive diagnosis in unaffected fetuses.

## Data Availability

All data generated or analysed during this study are included in this published article.
